# Knockdown of NCK1-AS1 inhibits the development of atherosclerosis by targeting miR-1197/COX10 axis

**DOI:** 10.1186/s13036-021-00281-6

**Published:** 2022-01-05

**Authors:** Bin Zhang, Juncheng Wang, Lei Du, Lufei Shao, Yourui Zou, Haibo Liu, Jinfang Liu

**Affiliations:** 1 Department of Neurosurgery, General Hospital of Ningxia Medical University, No.804 Shengli Street, Xingqing District, Yinchuan City, Ningxia Hui Autonomous Region 750004 PR China; 2 Department of Neurosurgery, People’s Hospital of Ningxia Hui Autonomous Region, Yinchuan City, Ningxia Hui Autonomous Region 750004 PR China

**Keywords:** Atherosclerosis, NCK1-AS1, miR-1197, COX10

## Abstract

**Background:**

Although long non-coding RNA (lncRNA) NCK1-AS1 plays important roles in human cancer, its function in atherosclerosis (AS) remains unclear.

**Method:**

The expression of NCK1-AS1 in AS blood samples was detected by qRT-PCR. Oxidized low-density lipoprotein (ox-LDL) was used to construct the AS cell model, and quantitative real-time polymerase chain reaction (qRT-PCR) assay was used to evaluate NCK1-AS1 level. Cell phenotypes including proliferation and apoptosis were assessed by Cell Counting Kit-8 (CCK-8) assay and flow cytometer, respectively. The malondialdehyde level was measured to evaluate oxidative stress. The expression of apoptosis-related proteins was evaluated by western blot. The expression of inflammatory cytokines (IL-1β, IL-6 and TNK-α) was measured by qRT-PCR and ELISA assays. The relationship among NCK1-AS1, miR-1197 and COX10 was determined by bioinformatic analysis and luciferase reporter assay.

**Results:**

NCK1-AS1 was significantly upregulated in AS blood samples and ox-LDL stimulated vascular smooth muscle cells (VSMCs). Knockdown of NCK1-AS1 increased cell viability, reduced cell apoptosis and MDA level, and also inhibited the expression of inflammatory cytokines (IL-1β, IL-6 and TNK-α) in ox-LDL stimulated VSMCs. NCK1-AS1 could positively regulate COX10 expression by directly sponging miR-1197. Moreover, co-transfection of sh-NCK1-AS1 and miR-1197 inhibitor, or co-transfection of sh-NCK1-AS1 and pc-COX10 (COX10 overexpressing plasmid) obviously reduced cell viability, promoted cell apoptosis, and increased MDA level in VSMCs followed by ox-LDL treatment for 24 h compared to that in sh-NCK1-AS1 transfected VSMCs.

**Conclusion:**

Our study revealed that knockdown of NCK1-AS1 attenuated the development of AS by regulating miR-1197/COX10 axis, suggesting that this lncRNA might be a potential therapeutic target for AS.

## Introduction

Atherosclerosis (AS) is a common leading cause of disability and death worldwide, which is closely involved in inflammation [1]. The major reason for this disease is the accumulation of low-density lipoprotein (LDL) in focal areas of medium and large arteries [2]. Recently, oxidized low-density lipoprotein (LDL) was used to construct the AS cell model in vitro [3]. Hence, more mechanisms involved in ox-LDL induced vascular smooth muscle cell (VSMC) injury is helpful to develop novel therapeutic targets for AS.

Long non-coding RNAs (lncRNAs) are a group of non-coding RNAs (> 200 nucleotide), and can participate in diverse biological processes [4]. Increasing evidences revealed that lncRNAs played essential roles in AS development through regulating the proliferation, apoptosis and inflammation of smooth muscle and endothelial cells [5, 6]. LncRNA NCK1 antisense RNA 1 (NCK1-AS1) is a newly identified lncRNA, and encodes a noncoding RNA with 1.4 kb in length [7]. Recently, NCK1-AS1 has been reported to play important roles in human cancers such as gastric cancer [8], lung squamous cell carcinoma [9], non-small cell lung cancer [10], urinary bladder cancer [11], ovarian cancer [12], and so on. However, the role of NCK1-AS1 in AS progression remains unclear.

MicroRNAs (miRNAs), is an another key non-coding RNA mediator that participated in biological pathways including AS [13]. MiRNAs have been identified to regulate the expression of downstream genes by binding to their 3′UTR and then inhibiting their expression [14]. Although the functions of miR-1197 in various biological processes including cell survival and apoptosis have been well studied [15], its mechanisms in ox-LDL-stimulated VSMCs remains unknown.

Cytochrome c oxidase assembly protein (COX10), a farnesyl transferase and is necessary for the maturation and stability of COX1, one of important cytokines with a pro-inflammatory function [16, 17]. It has been reported that high level of COX1 is positively correlated to the stronger inflammatory response during AS development [18]. In this study, we studied the role of NCK1-AS1/miR-1197/COX10 in cell proliferation, apoptosis and inflammatory response in ox-LDL stimulated VSMCs, and suggested that NCK1-AS1 might be a potential therapeutic target for AS.

## Materials and methods

### Clinical samples

Total 32 AS patients and 32 healthy volunteers were recruited at General Hospital of Ningxia Medical University between 2015.5 to 2020.9. Inclusion criteria: all patients were diagnosed with carotid atherosclerosis by angiography. Exclusion criteria: patients accompanied with cancers and autoimmune or inflammatory disease. The clinical features of participants were shown in Table 1. All participants have signed the written informed consent forms. The blood samples of all participants were collected and stored at − 80 °C immediately. This study was approved by the human Ethics Committee of General Hospital of Ningxia Medical University.

**Table 1:** Demographic, clinical and biochemical characteristics of study subjects

	Healthy	AS
Age (years)	*51.5* *+* *8.5*	*53.7* *+* *9.5*
Male/Female (n/n)	16/16	18/14
Total cholesterol (mmol/L)	*4.45* *+* *0.5*	4.8 + 1.35
Low density lipoprotein (mmol/L)	2.6 + 0.35	3.1 + 1.10
High density lipoprotein (mmol/L)	1.31 + 0.15	1 + 0.32
Total triglyceride (mmol/L)	0.98 + 0.35	1.6 + 0.88
Diabetes mellitus (n)	5	18
Serum creatine (mmol/L)	45.6 + 12.4	48.9 + 9.5
MDRD (ml/min per1.75 m^2)	87.5 + 5	90.25 + 7.8
NT-proBNP (pg/ml)	53.5 + 15.4	60.6 + 10.5
Aspirin (n)	2	10
Statins (n)	0	24

### Cell culture and treatment

Human vascular smooth muscle cells (VSMCs) were purchased from American type culture collection (ATCC; Manassas, VA, USA). and cultured in Dulbecco’s Modified Eagles Medium (DMEM, Invitrogen, California, USA) containing 10% fetal calf serum (FBS, Gibco, USA) in a 37 °C incubator with 5% CO_2_. To mimic the atherosclerosis in condition in vitro, VSMCs were stimulated by 25, 50 and 100 μg/ml of ox-LDL (UnionBiol, Beijing, China) for 24 h, or 50 μg/ml ox-LDL for 6, 12, 24 and 48 h. To investigate the function of NCK1-AS1, transfected VSMCs were treated with 50 μg/ml ox-LDL for 24 h, and cells were used to perform subsequent experiments.

### Cell transfection

The short hairpin RNA targeting NCK1-AS1 (sh-NCK1-AS1), negative control sh-NC, miR-1197 mimics (miR mimics), mimics NC, miR-1197 inhibitor (miR inhibitor) and inhibitor NC were purchased from GenePharma (Shanghai, China). sh-NCK1-AS1: GAAUGUCAUCCCAGCCGAAT, and sh-NC: UUCUCCGAACGUGUCACGUTT. To overexpress COX10, pcDNA3.1-based recombinant-overexpressing plasmid specific to COX10 was also constructed by GenePharma (Shanghai, China), and the empty vector as the negative control. 30 nM sh-RNA/mimics/inhibitor and 100 ng recombinant-overexpressing vector were transfected into VSMCs by Lipofectamine 2000 (Invitrogen). After 48 h of transfection, cells were used for subsequent experiments.

### Cell proliferation

After transfection, VSMCs (2 × 10^5^ cells/well) were stimulated by 50 μg/ml ox-LDL for 24 h. Cell viability was assessed by incubating with 10 μl of Cell Counting Kit-8 (CCK-8, Dojindo Molecular Technologies) reagent for 4 h. The absorbance at 450 nm was detected by a microplate reader. The assay was performed in triplicates of biological replicates.

### Apoptosis analysis

After transfection, VSMCs (2 × 10^5^ cells/well) were stimulated by 50 μg/ml ox-LDL for 24 h, harvested, washed in PBS and suspended to 100 μl 1× binding buffer, followed by the incubated with 5 μl of fluorescein isothiocyanate (FITC)-labeled Annexin-V and 5 μl of propidium iodide (PI) for 15 min in the dark according to the manufacturer’s instruction (Cat. No. 556419, KeyGen Biotech). The apoptotic cells were analyzed by flow cytometer (BD Biosciences, Cowley, UK). The assay was performed in triplicates of biological replicates.

### Quantitative real time-polymerase chain reaction (qRT-PCR)

Total RNA was extracted by using TRIzol reagent. The cDNA was reverse transcribed from total RNAs by PrimeScript™ RT Master Mix (TaKaRa, Beijing, China) and the reverse transcription reactions were conducted on a LightCycler 480 (Roche Diagnostic, Sussex, UK) using 2 x SYBR Green qPCR Master Mix (Absource, Munich, Germany). The assay was performed in triplicates of biological replicates. The relative expression of target genes was calculated by the 2^−ΔΔCt^ method, with glyceraldehyde-3-phosphate dehydrogenase (GAPDH) and U6 small nuclear RNA (U6) as the internal reference. The primers were listed in Table 2.

**Table 2:** The sequences of specific primers

Gene name	Primer sequence (5′ to3’)
NCK1-AS1	Forward: 5′-AGTTCAGCCCCCACTGCTCT-3′ Reverse: 5′-TGGTTTGAGTTCCCATTTCTC-3’
COX10	Forward: 5’-TCTGTTGTGGCTGGCTTTGGAC-3′ Reverse: 5′-CTTCTCTGGCAATTCTTTCCTGG-3’
IL-1β	Forward: 5’-CCACAGACCTTCCAGGAGAATG-3′ Reverse: 5′-GTGCAGTTCAGTGATCGTACAGG-3’
IL-6	Forward: 5’-AGACAGCCACTCACCTCTTCAG-3′ Reverse: 5′-TTCTGCCAGTGCCTCTTTGCTG-3’
TNF-a	Forward: 5’-CTCTTCTGCCTGCTGCACTTTG-3′ Reverse: 5′-ATGGGCTACAGGCTTGTCACTC-3’
GAPDH	Forward: 5’-ATCCACGGGAGAGCGACAT-3′ Reverse: 5′-CAGCTGCTTGTAAAGTGGAC-3’
U6	Forward: 5’-ACAGATCTGTCGGTGTGGCAC-3′ Reverse: 5′-GGCCCCGGATTATCCGACATTC-3’

### Western blot

Total protein was extracted by using RIPA lysis buffer (Sigma-Aldrich). Approximately equal amounts of protein were separated by 10% SDS-PAGE gels and then transferred into PVDF membranes (Roche, Basel, Switzerland). After blocking with 5% non-fat dry milk for 1 h, the membranes were incubated with primary antibodies against COX10 (1: 1000, ab228734), caspase 3 (1:1000, ab32351), cleaved-caspase 3 (1:1000, ab32042), Bax (1:1000, ab32503), Bcl-2 (1:1000, ab32124), and GAPDH (1:10000, ab181602) overnight at 4 °C. Then the membranes were incubated with HRP-conjugated secondary antibodies (1:10,000) for 2 h. All antibodies were purchased from Abcam. Protein bands were detected by enhanced chemiluminescence kit. The proteins were quantified by Quantity One software (Bio-Rad Laboratories, Inc., Hercules, CA, USA). The assay was performed in triplicates of biological replicates.

### Target prediction

The binding sites among NCK1-AS1, miR-1197 and COX10 were predicted by Starbase (http://starbase.sysu.edu.cn/index.php) and TargetScan (http://www.targetscan.org/), respectively as previously described [19].

### Luciferase reporter assay

The full fragment of NCK1-AS1 and 3′UTR of COX10 containing the wild type (WT) or mutant type (MUT) miR-1197 binding site were synthesized and cloned into pmirGLO vector (Promega, Mannheim, Germany) to generate recombinant luciferase vectors (NCK1-AS1 WT, NCK1-AS1 MUT, COX10 WT and COX10 MUT). The recombinant luciferase reporter vector was co-transfected singly with miR mimics or mimics NC into VSMCs using Lipofectamine 2000. Finally, a dual luciferase reporter assay system (Promega, USA) was used to detect the relative luciferase activities. The relative firefly luciferase activity was calculated by normalizing to *renilla* luciferase activity. The assay was performed in triplicates of biological replicates.

### Malondialdehyde (MDA) content

To evaluate the oxidative stress, transfected VSMCs (2 × 10^5^ cells/well) were stimulated by 50 μg/ml ox-LDL for 24 h. Subsequently, cells were harvested, lysed and the malondialdehyde (MDA) content was measured by using Lipid Peroxidation MDA Assay kit (cat. no. S0131, Beyotime) according to the manufacturer’s instructions. The oxidative stress was presented as the percentage of control cells without any treatments. The assay was performed in triplicates of biological replicates.

### ELISA assay

To evaluate the effect of NCK1-AS1 on inflammatory response, transfected VSMCs (2 × 10^5^ cells/well) were treated with 50 μg/ml ox-LDL for 24 h. Cells were lysed, and the production of inflammatory cytokines (IL-1β, IL-6 and TNK-α) were detected by using the commercial detection kits (Thermo Fisher Scientific, USA), respectively. The assay was performed in triplicates of biological replicates.

### Statistical analysis

All data were presented as mean ± standard deviation (SD), and statistical analysis was performed using SPSS 18.0 software. Difference was tested by using student’s t test (two groups) and one-way analysis of variance (ANOVA) (multiple groups). *P* < 0.05 was considered statistically significant.

## Results

### NCK1-AS1 was highly expressed during AS development

**Fig. 1:**
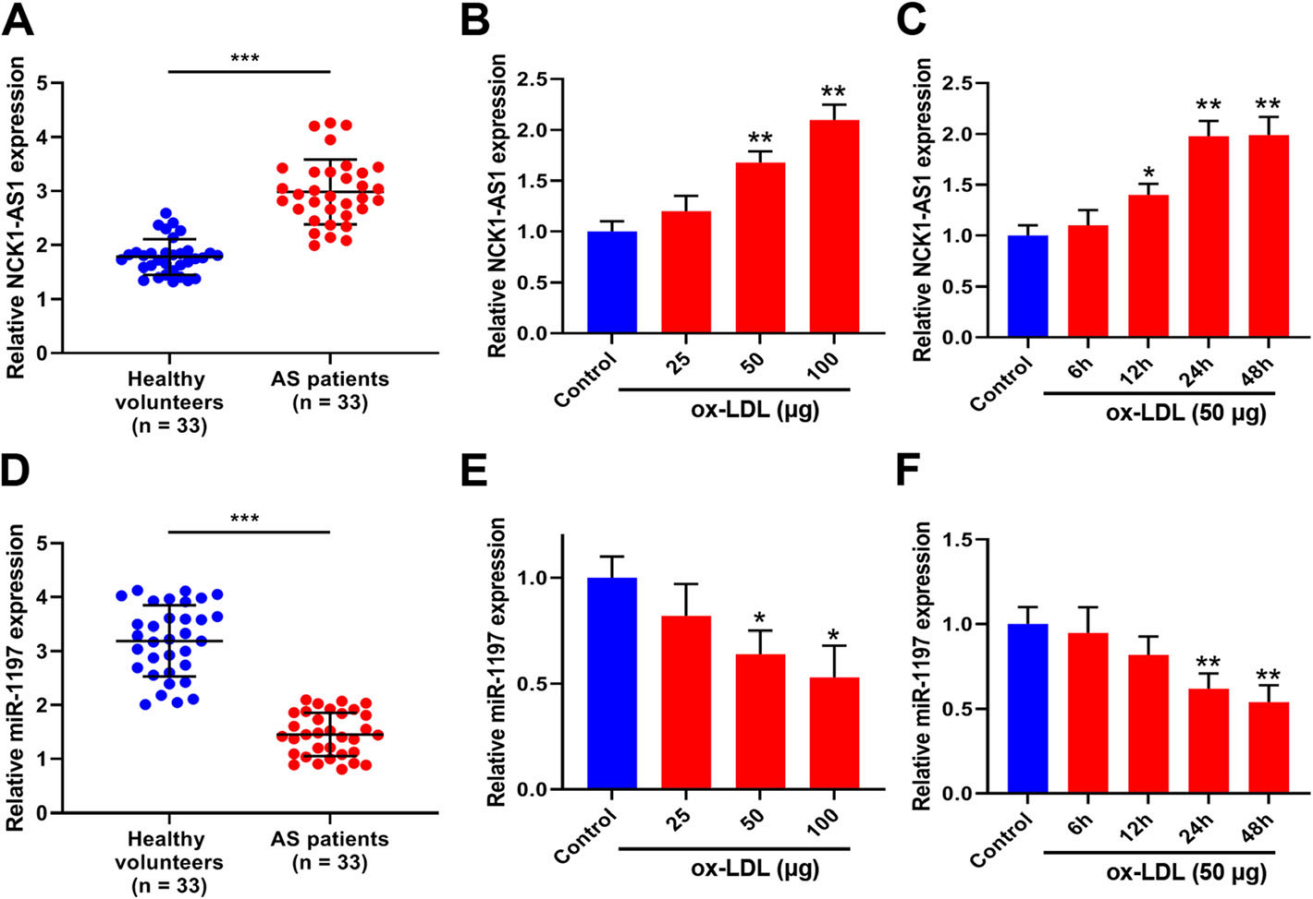
The expression of NCK1-AS1 and miR-1197 during AS progression. **A** The expression of NCK1-AS1 in blood samples of AS patients was detected by qRT-PCR (*n* = 32). **B** and **C** VSMCs were stimulated by 25, 50 and 50 μg/ml ox-LDL for 24 h **B** or 50 μg/ml ox-LDL for 6, 12, 24 and 48 h **C**. The expression of NCK1-AS1 was detected by qRT-PCR. **D** The expression of miR-1197 in blood samples of AS patients was detected by qRT-PCR (n = 32). (E and F) VSMCs were stimulated by 25, 50 and 50 μg/ml ox-LDL for 24 h **E** or 50 μg/ml ox-LDL for 6, 12, 24 and 48 h **F**. The expression of miR-1197 was detected by qRT-PCR. * *p* < 0.05, ** *p* < 0.01 and *** *p* < 0.001

To investigate the role of NCK1-AS1 in AS, we firstly detected the expression of NCK1-AS1 and miR-1197 in the blood samples of AS patients and ox-LDL treated VSMCs. The results showed that NCK1-AS1 level increased 1.66 fold in the blood samples of AS patients compared to that in healthy volunteers (Fig. 1A), also upregulated 1.51 fold and 2.08 fold in 50 μg/ml ox-LDL treated VSMCs and 100 μg/ml ox-LDL VSMCs for 24 h compared to that in control cells, respectively (Fig. 1B). In addition, NCKA-AS1 level was also regulated 1.41 fold, 1.79 fold and 1.78 fold in 50 μg/ml ox-LDL treated VSMCs for 12 h, 24 h and 48 h, respectively (Fig. 1C). For miR-1197 expression, is was downregulated 2.13 fold in the blood samples of AS patients compared to that in healthy volunteers (Fig. 1D). Also, miR-1197 level was downregulated 1.42 fold and 2.13 fold in 50 μg/ml ox-LDL treated VSMCs and 100 μg/ml ox-LDL VSMCs for 24 h compared to that in control cells, respectively (Fig. 1E). MiR-1197 level was also downregulated 1.53 fold and 1.64 fold in 50 μg/ml ox-LDL treated VSMCs for 24 h and 48 h, respectively (Fig. 1F). Then 50 μg/ml ox-LDL for 24 h was used for the subsequent experiments.

### Knockdown of NCK1-AS1 increased cell viability, reduced oxidative stress and promoted cell apoptosis in ox-LDL stimulated VSMCs

**Fig. 2:**
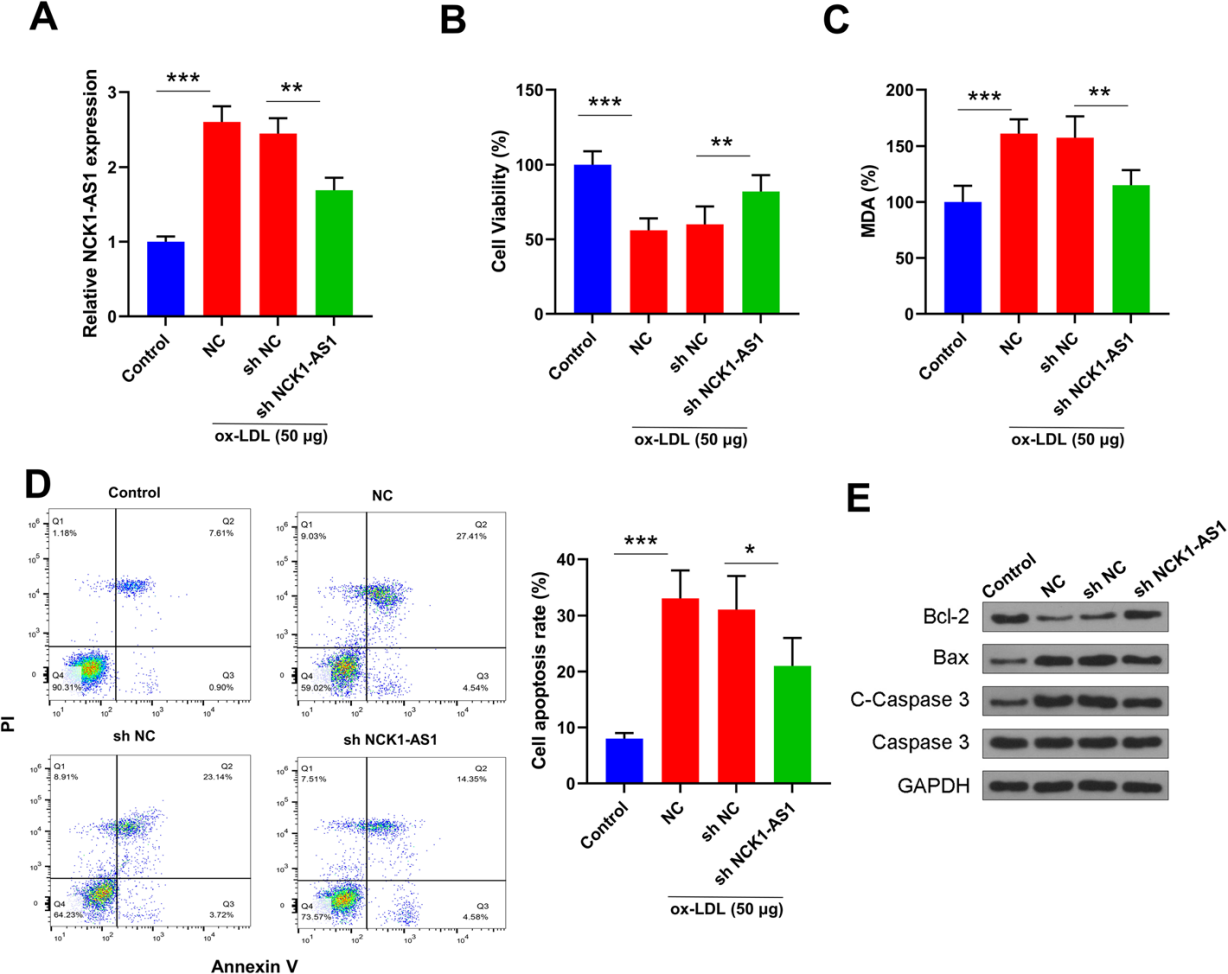
Knockdown of NCK1-AS1 attenuated ox-LDL induced cell injury in VSMCs. VSMCs were transfected with sh-NCK1-AS1 or sh-NC, and then treated with 50 μg/ml ox-LDL for 24 h. **A** The expression of NCK1-AS1 was detected by qRT-PCR. **B** CCK-8 assay. **C** MDA content was measured by specific detection kit. **D** Flow cytometry for cell apoptosis. **E** The expression of cleaved caspase3, Bax and Bcl-2 was measured by western blot. Control group is that VSMCs without any treatment, NC means that VSMCs received ox-LDL treatment. * p < 0.05, ** p < 0.01 and *** *p* < 0.001

To evaluate the role of NCK1-AS1, sh-NCL-AS1 was transfected into VSMCs to knockdown it. The results of qRT-PCR showed that sh-NCK1-AS1 reduced the level of NCK1-AS1 (1.62 fold) in ox-LDL treated VSMCs compared with sh-NC group and NC group (Fig. 2A). ox-LDL treatment decreased cell viability (2.16 fold), increased MDA content (1.67 fold) and promoted cell apoptosis (4.15 fold) of VSMCs compared to that in control group, and knockdown of NCK1-AS1 increased cell viability (1.45 fold), reduced MDA content (1.61 fold) and cell apoptosis (1.42 fold) in ox-LDL stimulated VSMCs compared with sh-NC group and NC group (Fig. 2B-D). In addition, ox-LDL increased the protein expression of apoptosis-related marker cleaved caspase 3 and pro-apoptotic factor Bax, while reduced anti-apoptotic factor Bcl-2 level in VSMCs compared with control group, and knockdown of NCK1-AS1 reduced the expression of cleaved caspase 3 and Bax, while increased Bcl-2 expression compared with sh-NC and NC group in ox-LDL treated VSMCs (Fig. 2E). The results suggested that knockdown of NCK1-AS1 inhibited AS development in vitro.

### NCK1-AS1 regulated COX10 by sponging miR-1197

**Fig. 3:**
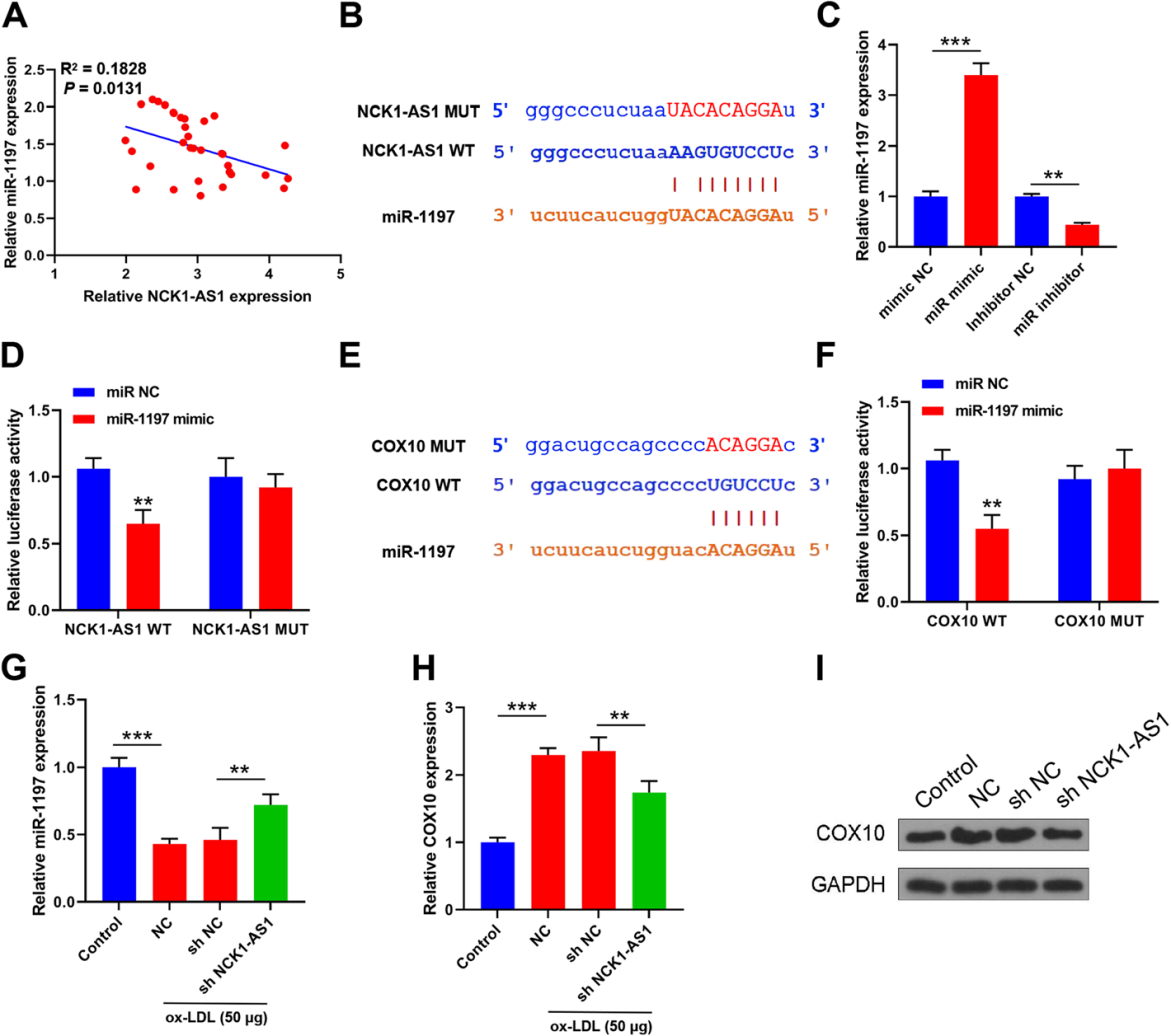
NCK1-AS1 regulated COX10 by sponging miR-1197. **A** Correlation between NCK1-AS1 and miR-135a-5p level in AS blood samples. **B** Schematic model of miR-1197 binding site within NCK1-AS1 predicted by Starbase and the construction of mutant miR-1197 binding site. **C** VSMCs were transfected with miR-1197 mimics or inhibitor, and qRT-PCR for miR-1197 level. **D** VSMCs were transfected with miR-1197 mimics or mimics NC, and the relative luciferase activity. **E** Schematic model of miR-1197 binding site within 3′UTR COX10 predicted by Targetscan and the construction of mutant miR-1197 binding site. **F** VSMCs were transfected with miR-1197 mimics or mimics NC, and the relative luciferase activity. **G - I** VSMCs were transfected with sh-NCK1-AS1, and then treated with 50 μg/ml ox-LDL for 24 h. QRT-PCR for miR-1197 level **G**, COX10 level **H**, and western blot for COX10 level **I**. * p < 0.05, ** p < 0.01, and *** p < 0.001

Due to the negative correlation between the level of NCK1-AS1 and miR-1197 in the blood samples of AS patients (Fig. 3A), we thought that NCK1-AS1 might serve as the sponge of miR-1197. Starbase prediction showed that there was a putative binding site between NCK1-AS1 and miR-1197, and a mutant sequence of NCK1-AS1 against miR-1197 binding site was constructed (Fig. 3B). Overexpression of miR-1197 increased the expression of miR-1197 (3.47 fold) compared with mimics NC group, and miR-1197 inhibitor reduced miR-1197 expression (2.24 fold) compared with inhibitor NC group (Fig. 3C). Luciferase reporter assay indicated that miR-1197 mimics reduced the luciferase activity of NCK1-AS1 WT (1.64 fold) compared with miR-NC group (Fig. 3D). Meanwhile, the results of Targetscan prediction suggested that COX10 was a target of miR-1197, and a mutant sequence of 3′UTR COX10 against miR-1197 binding site was constructed (Fig. 3E). Luciferase reporter assay also confirmed the binding relationship between miR-1197 and 3′UTR of COX10 (1.58 fold) (Fig. 3F). Moreover, ox-LDL caused a significant downregulation of miR-1197 compared with control group (2.17 fold), and knockdown of NCK1-AS1 increased miR-1197 expression (1.46 fold) compared with sh-NC in ox-LDL treated VSMCs (Fig. 3G). In addition, COX10 was notably upregulated (2.34 fold) in ox-LDL treated VSMCs, and knockdown of NCK-1AS1 reduced the expression of COX10 (1.33 fold) compared with sh-NC in ox-LDL treated VSMCs (Fig. 3H and I). These results indicated that NCK1-AS1 could regulate COX10 by sponging miR-1197 in ox-LDL treated VSMCs.

### Knockdown of NCK1-AS1 attenuated ox-LDL-induced VSMCs injury through regulating miR-1197/COX10 axis

**Fig. 4:**
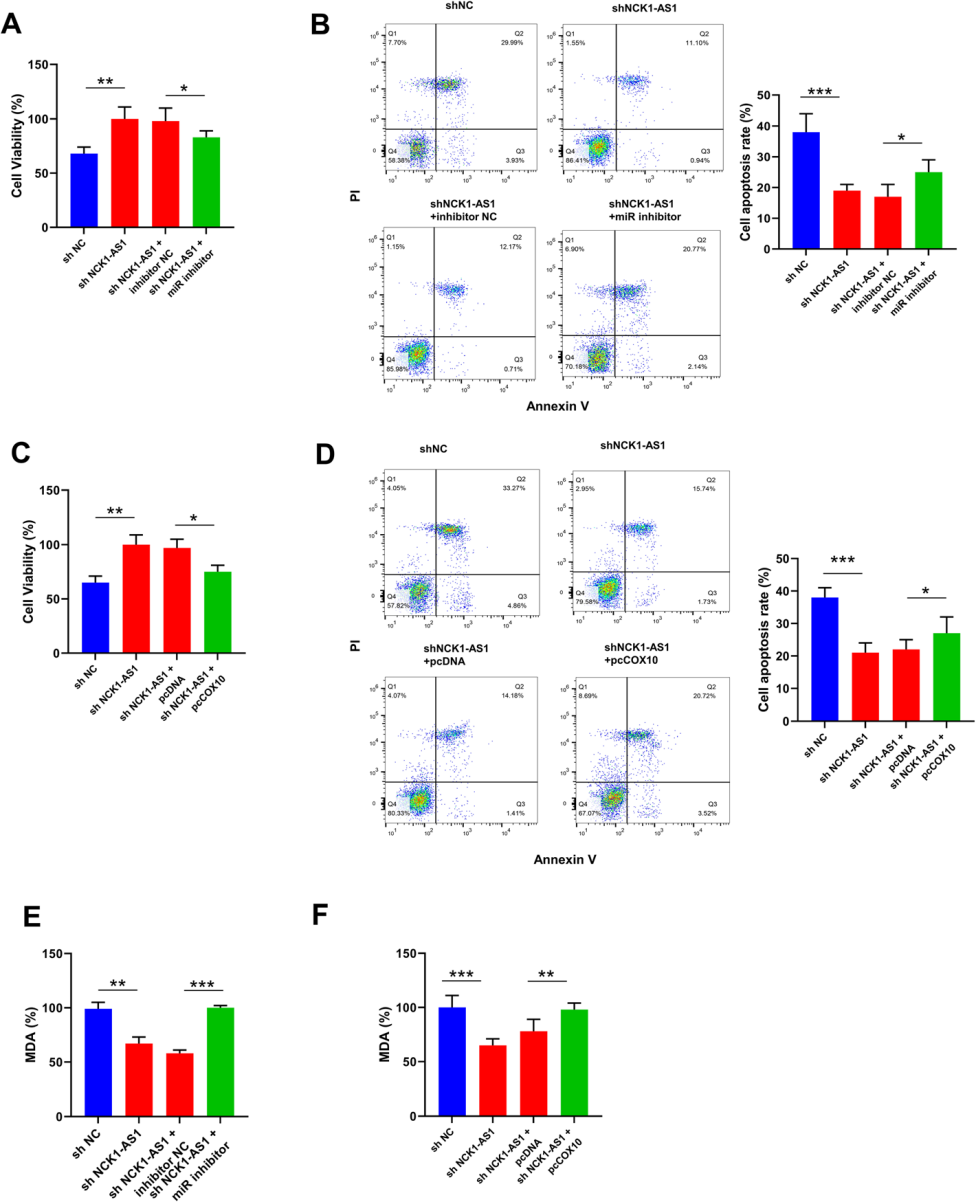
Knockdown of NCK1-AS1 attenuated ox-LDL-induced VSMCs injury through regulating miR-1197/COX10 axis. **A** and **B** CCK-8 assay for cell viability **A** and flow cytometry for cell apoptosis **B** after co-transfection of sh-NCK1-AS1 and miR-1197 inhibitor in VSMCs followed by 50 μg/ml ox-LDL for 24 h. **C** and **D** CCK-8 assay for cell viability **C** and flow cytometry for cell apoptosis **D** after co-transfection of sh-NCK1-AS1 and pc-COX10 (COX10 overexpressing plasmid) in VSMCs followed by 50 μg/ml ox-LDL for 24 h. **E** and **F** VSMCs were co-transfected with sh-NCK1-AS1 and miR-1197 inhibitor **E**, or sh-NCK1-AS1 and pc-COX10 **F**, and then treated by 50 μg/ml ox-LDL for 24 h. The MDA content was measured by specific detection kit. * p < 0.05, ** p < 0.01, and *** p < 0.001

To determine the function of NCK1-AS1/miR-1197/COX10 axis in AS, the rescue experiments were performed. Co-transfection of miR-1197 inhibitor and sh-NCK1-AS1 attenuated the elevation of cell viability (1.25 fold) and the reduction of cell apoptosis (1.39 fold) in sh-NCK1-AS1 transfected VSMCs followed by ox-LDL treatment for 24 h (Fig. 4A and B). Meanwhile, co-transfection of pc-COX10 (COX10 overexpressing plasmid) and sh-NCK1-AS1 attenuated the elevation of cell viability (1.67 fold) and the reduction of cell apoptosis (1.34 fold) in sh-NCK1-AS1 transfected VSMCs followed by ox-LDL treatment for 24 h (Fig. 4C and D). In addition, both co-transfection of sh-NCK1-AS1 and miR-1197 inhibitor, as well as co-transfection of sh-NCK1-AS1 and pc-COX10 notably increased MDA content in sh-NCK1-AS1 transfected VSMCs followed by ox-LDL treatment for 24 h (Fig. 4E and F). These findings revealed that knockdown of NCK1-AS1 attenuated ox-LDL-induced VSMCs injury through regulating miR-1197/COX10 axis.

### Knockdown of NCK1-AS1 attenuated inflammatory response in ox-LDL-induced VSMCs

**Fig. 5:**
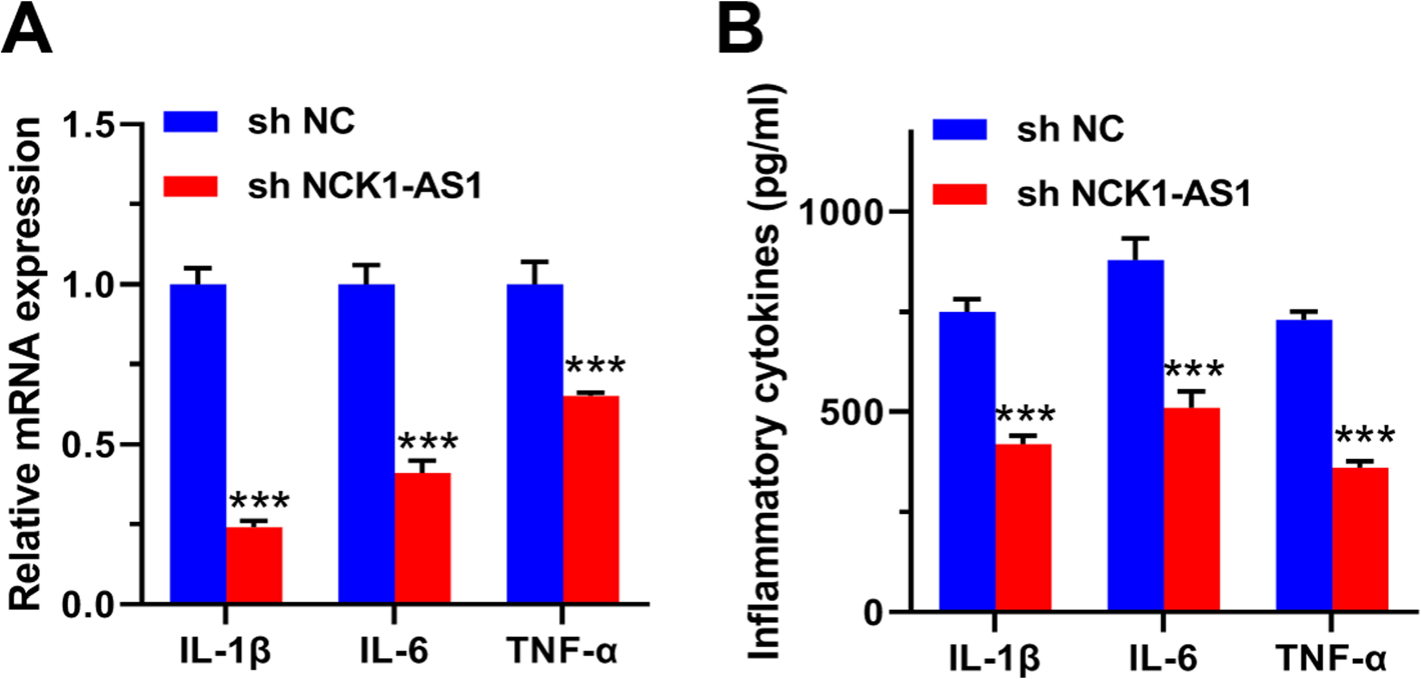
Knockdown of NCK1-AS1 attenuated inflammatory response in ox-LDL-induced VSMCs. VSMCs were transfected with sh-NCK1-AS1 or sh-NC, and then treated with 50 μg/ml ox-LDL for 24 h. The expression of inflammatory cytokines (IL-1β, IL-6 and TNK-α) was evaluated by qRT-PCR **A** and ELISA assay **B**. *** p < 0.001

Finally, we evaluated the impacts of NCK1-AS1 on inflammatory response in AS, and found that knockdown of NCK1-AS1 reduced the expression of inflammatory cytokines (IL-1β, 2.04 fold; IL-6, 1.19 fold and TNK-α, 1.17 fold) compared with sh-NC in ox-LDL stimulated VSMCs (Fig. 5A). In addition, knockdown of NCK1-AS1 reduced the production of inflammatory cytokines (IL-1β, 1.53 fold; IL-6, 1.71 fold and TNK-α, 1.62 fold) compared with sh-NC in ox-LDL stimulated VSMCs (Fig. 5B). These data further confirmed the inhibitory action of NCK1-AS1 knockdown on inflammatory response in AS progression.

## Discussion

In this study, we investigated the regulatory mechanism of NCK1-AS1/miR-1197/COX10 axis in the progression of AS, and our results constructed a AS cell model using ox-LDL to stimulate VSMCs, and found that ox-LDL notably reduced cell viability, increased cell apoptosis and inflammatory response, which was consistent with previous findings [20, 21]. These data confirmed our successful AS cell model, which was subsequently used to investigate the role of NCK1-AS1 during AS development.

Interestingly, we found that NCK1-AS1 was significantly upregulated in AS blood samples and ox-LDL treated VSMCs. Moreover, knockdown of NCK1-AS1 obviously increased cell viability, reduced cell apoptosis and inflammatory response in ox-LDL treated VSMCs. Previous studies have reported the essential functions of NCK1-AS1 in other human diseases. For example, inhibition of NCK1-AS1 prevents the migratory and invasive capacities in nasopharyngeal carcinoma cells [22]. Knockdown of NCK1-AS1 inhibits cell proliferative rate and migratory ability via the suppression of miR-134 in cervical cancer [23]. These reports indicated that NCK1-AS1 played important functions in cell survival and apoptosis. In this study, we revealed the essential role of NCK1-AS1 in cell proliferation, apoptosis and inflammatory response in ox-LDL stimulated VSMCs, suggesting that NCK1-AS1 might be a novel diagnostic and therapeutic biomarker for AS.

More previous studies revealed that lncRNAs can function as the sponge of miRNAs to participate in the various pathogenic processes [24]. For instance, SNHG7–003 suppresses cell proliferation and promoted apoptosis of VSMCs by targeting miR-1306-5p [25]. Kcnq1ot1 exacerbates the lipid accumulation and promotes AS progression through functioning as the competing endogenous RNAs (ceRNAs) of miR-452-3p [26]. Here, we identified that miR-1197 was negatively regulated by NCK1-AS1, and luciferase reporter assay and function assays confirmed this interaction relationship. Moreover, downregulation of miR-1197 obviously reversed the protective impacts of NCK1-AS1 knockdown in ox-LDL stimulated VSMCs injury. However, there were other previously identified miRNA targeted by NCK1-AS1 including miR-137 [27], miR-22-3p [8], miR-22-3p [28], and more attentions should be focused on these potential NCK1-AS1-downstream miRNAs regulatory axis during AS development.

In addition, our results also identified that COX10 was a direct target of miR-1197, and miR-1197 could directly bind to its 3′UTR and then inhibit COX10 expression. It has been reported that miRNAs can regulate the progression of AS by targeting downstream target genes [29]. Although inflammatory response always occurs during AS progression, the function of COX10 in AS remains unclear. In this study, we found that knockdown of NCK1-AS1 significantly reduced the expression of COX10 in ox-LDL treated VSMCs, and overexpression of COX10 obviously eliminated the protective roles of NCK1-AS1 knockdown on cell proliferation, apoptosis and inflammatory response. These results further determined that COX10 participated in the regulatory function of NCK1-AS1/miR-1197 in AS development. Similarly, miRNAs can regulate a series of downstream target genes in human diseases. For example, miR-1197 controls the progression of non-small-cell lung carcinoma through regulating MADD [30], and also promotes cell proliferation by upregulating HOXC11 [31]. Hence, there might be other potential target genes that mediated the function of miR-1197 in AS development, and this conjecture needed to be further investigated in the subsequent experiments.

In conclusion, our study demonstrated that knockdown of NCK1-AS1 could efficiently attenuate ox-LDL induced VSMCs injury through regulating miR-1197 and COX10, providing that NCK1-AS1 might be a potential therapeutic target for AS.

## Data Availability

The data that support the findings of this study are available on request from the corresponding author.
